# Genetic analysis of genome sequence characteristics of two lumpy skin disease viruses isolated from China

**DOI:** 10.1186/s12917-022-03525-9

**Published:** 2022-12-07

**Authors:** Lin Li, Zhenzhong Wang, Chuanxiang Qi, Shan Liu, Mingxia Gong, Jinming Li, Xiaodong Wu, Zhiliang Wang

**Affiliations:** 1 China Animal Health and Epidemiology Center, Qingdao, 266032 Shandong China; 2 MOE Joint International Research Laboratory for Animal Health and Food Safety/Nanjing Agricultural University, Nanjing, 210095 Jiangsu China

**Keywords:** China, Genome sequence, Genetic analysis, LSDV

## Abstract

**Background:**

Lumpy skin disease (LSD) is an acute or subacute infectious disease caused by lumpy skin disease virus (LSDV) of genus *Capripoxvirus*. The outbreaks of LSD were confirmed in the Yili area of the Xinjiang autonomous region in August 2019 and the Fujian province in June 2020. We detected LSDV in our daily monitoring work, then isolated, identified and sequenced the virus, and analyzed the whole genome characteristics of the isolated strain.

**Results:**

Whole genome sequencing revealed that the strains isolated were all LSDV and were named as LSDV XJ201901 and LSDV FJ2019. The results showed that the identity based on whole genome sequences between LSDV XJ201901 and LSDV FJ2019 was 100% and the identity based on whole genome sequences between the two isolated strains and the global LSDV strains was 97.28%-99.99%, with the strain LSDV72/PrachuapKhiriKhan/Thailand/2021 (99.99%) having the highest sequence identity. Analysis of potential recombination events revealed that a total of 18 potential recombination events were identified in strains LSDV XJ201901 and LSDV FJ2019. The two strains are a recombination of Neethling vaccine LW 1959 (GeneBank: AF409138.1) with KSGP 0240 (GeneBank: KX683219.1). It was observed that Neethling vaccine LW 1959 (11/18) and KSGP 0240 (10/18) are involved in most of the potential recombination events.

**Conclusions:**

The virus isolate in this study was LSDV and was identified as a vaccine recombinant strain. The most likely potential parent strains of the two strains in this study are Neethling vaccine LW 1959 and KSGP 0240. The strains in this study are very similar to those isolated in East and Southeast Asia since 2019.

## Background

Lumpy skin disease (LSD) is an acute or subacute infectious disease caused by lumpy skin disease virus (LSDV) of genus *Capripoxvirus in Poxviridae*. Cattle are the natural host of LSDV, and all kinds of cattle are susceptible to LSDV [1]. LSDV infection can lead to weight loss, significant reduction of milk production and even death in cattle. The mortality rate of cattle infected with LSDV was up to 10% and even 20%, causing serious economic losses and affecting international trade [2]. Water buffalos [3], antelope [4] and giraffe can also be infected with LSDV. LSD is a notifiable disease stipulated by the World Organization for Animal Health (OIE, 2017) and 1 of the 15 class I infectious diseases stipulated by List of imported animal quarantine diseases of the China. LSD was first observed in 1929 in Zambia in East Africa [5], from where it spread across the African continent and beyond the Sahara Desert. LSD spread to Israel in the Middle East in 1989 [6]. It continued to be prevalent in Africa and the Middle East in the following 30 years, with local epidemics in some regions. The outbreak of LSD was reported in Greece, Turkey, Russia and Eastern Europe in 2015 [7–10], and spread to Bulgaria, Macedonia, Serbia, Niger, Albania, Montenegro, Nigeria and other countries a year later [10]. From 2017 to 2018, LSD continued to move eastward along the border between Russia and Kazakhstan and gradually approached Xinjiang in China. The risk of LSDV being introduced into China is great, which poses a serious threat to China [11]. In August 2019, China reported the first case of LSD epidemic, which occurred in Yili, Xinjiang [12]. Since then, other provinces in western and eastern China have reported LSD outbreaks. LSD has become a new threat to China's cattle industry.

LSDV is a double stranded DNA virus with a genome size of 145-156kb. It is composed of the central core coding region and the same inverted terminal repeat (ITR) with a length of 2.4kb on both sides. The GC content in LSDV genome was only 27% [13], showing a regular and uniform distribution. The LSDV genome contains 156 ORFs encoding proteins of 53 to 2,027 amino acids [14]. LSDV has high genomic identity with goatpox virus (GTPV)and sheeppox virus (SPPV), more than 98%. The protein encoded by ORF36 (gene RPO30) plays a role in DNA-directed 5'-3' RNA polymerase activity. The GPCR protein encoded by ORF11 (gene GPCR) is a predicted chemockine receptor, which plays a role in the tropism of the virus host. Both of these two genes are frequently used for homology analysis and molecular biological detection between different strains [15]. However, there are only a few methods to identify and detect three poxviruses of the genus *Capripoxvirus* due to the high sequence identity of LSDV, GPTV and SPPV. The methods of distinguishing between the tree *Capripoxviruses* are based on genetic characterizations [16].

The prevention and control of LSD mainly rely on live attenuated vaccine for immunization in LSD endemic countries. Many attenuated strains including cell passage attenuated strains and genetically modified attenuated strains, such as LSDV Neethling, KSGP O-240 and LSDV_WB005KO, were used for the prevention and control of LSDV. Although LSD vaccine is widely used to control the outbreak of epidemic diseases, the reinfection of immunized animals still exists [17].

Since 2019, at least seven provinces have reported LSD outbreaks in China according to publicly available official data [12] (http://www.xmsyj.moa.gov.cn/yqfb/202007/t20200715_6348686.htm). Several LSDV strains have been isolated and reported in China until 2022. We detected LSDV in our daily monitoring work, then isolated, identified and sequenced the virus, and analyzed the whole genome characteristics of the isolated strain.

## Results

### Virus isolation and identification

The disease material suspected of being infected with LSDV was homogenized and centrifuged, and the supernatant was inoculated with LT cells in order to isolate the virus. The cells were observed day by day onto a microscope until CPE was observed. LT cells contracted and became round, and a large number of cytopathic effect (CPE) appeared after infection with LSDV (Fig. 2B). The cells were repeatedly frozen and thawed for 3 times after 6 days of virus infection and the total DNA was extracted for qPCR with primers and probe listed in Table 1. The results of qPCR showed that the isolated virus was LSDV (results not shown).

**Table 1:** Primers used for qPCR

Primer	Sequence
Forward primer	5’-TGAATTAGTGTTGTTTCTTC-3’
Reverse primer	5’-GGGAATCCTCAAGATAGTTCG-3’
Probe	5’-FAM-TGCCGCAAAATGTCGA-MGB-3’

### Whole genome sequencing and sequence analysis

Whole genome sequencing was performed using Illumina nextseq 500 and mapped to China/GD01/2020 (Genebank: MW355944). Two strains of LSDV involved in this study have been submitted to NCBI, one of which is named LSDV XJ201901 (Genebank: OM984485) and the other is named LSDV FJ2019 (Genebank: OM984486). The reference sequences were downloaded from NCBI and the sequence alignment was performed by Mega X. The results showed that the identity based on whole genome sequences between LSDV XJ201901 and LSDV FJ2019 was 100%. The identity based on whole genome sequences between the China strains and other reference sequences was 97.28%-99.99%, with the strain LSDV72/PrachuapKhiriKhan/Thailand/2021(GeneBank: ON152411.1) having the highest identity (Fig. 3A). In addition, two important genes RP030 and GPCR in LSDV were also used for identity analysis (Fig. 3B and C). The similarity between LSDV XJ201901 or LSDV FJ2019 and the global LSDV strains based on GPCR was 95.52%-100%. The strains with the highest identity with strains in this study based on GPCR are strains isolated in China, Vietnam and Thailand since 2019. The similarity between LSDV XJ201901 or LSDV FJ2019 and the global LSDV strains based on RP030 was 97.70%-100%. The strains with the highest identity with strains in this study based on RP030 are also strains isolated in China, Vietnam and Thailand since 2019.

All global LSDV strains of LSDV were divided into different groups according to the results of sequence alignment. The group of cluster 1.1, cluster 1.2 and the strains before 2019 were used to analyse the gene recombination events involved LSDV XJ201901 and LSDV FJ2019 using RDP4 and Simplot. 18 potential recombination events were predicted in LSDV XJ201901 and LSDV FJ2019 by RDP4 algorithm The potential strains involved in gene recombination events were shown in the Fig. 4 and Fig. 5. All strains involved in potential recombination events are listed in Table 2. Neethling vaccine LW 1959 (11/18) and KSGP 0240 (10/18) are involved in most of the potential recombination events by counting. There were 5 recombination events directly related to the two strains, which is the largest number of all forecast recombination.

**Table 2:** The isolates involved potential recombination events and P values

No.	Potential recombinant location		Potential major parent	Potential manor parent	*P*-Values					
	Start (bp)	Stop (bp)			RDP	GENECONV	Bootscan	Maxchi	Chimaera	SISscan
1	353	4762	LSDV/Russia/Saratov/2017	KSGP 0240	1.51E-21	1.19E-22	-	#######	#######	#######
2	7464	10380	Neethling vaccine LW 1959	LSDV/Russia/Dagestan/2015	1.96E-11	7.05E-09	#######	#######	#######	-
3	19655	23698	Neethling vaccine LW 1959	KSGP 0240	-	6.45E-12	#######	#######	#######	#######
4	27054	28070	LSDV/Russia/Saratov/2017	155920/2012	1.68E-06	1.14E-06	-	#######	#######	-
5	34306	35434	LSDV/Russia/Saratov/2017	KSGP 0240	1.68E-06	1.54E-07	#######	#######	-	-
6	45556	48925	Neethling vaccine LW 1959	KSGP 0240	-	1.57E-07	-	#######	#######	#######
7	48926	53919	Neethling vaccine LW 1959	LSDV/Russia/Dagestan/2015	7.61E-12	2.33E-07	#######	#######	#######	-
8	67176	84154	Neethling vaccine LW 1959	KSGP 0240	6.80E-34	2.83E-33	#######	#######	#######	#######
9	86026	89565	LSDV/Russia/Udmurtiya/2019	KSGP 0240	3.58E-15	1.93E-12	#######	#######	#######	#######
10	91714	93194	Neethling vaccine LW 1959	KSGP 0240	1.18E-10	6.24E-09	#######	#######	#######	-
11	95980	99978	Neethling vaccine LW 1959	KZ-Kostanay-2018	1.31E-19	3.67E-18	#######	#######	#######	-
12	102308	105101	LSDV/Russia/Saratov/2017	KSGP 0240	7.70E-09	5.11E-07	-	#######	-	-
13	108656	111780	LSDV/Russia/Saratov/2017	KSGP 0240	1.62E-11	2.74E-09	#######	#######	#######	-
14	119834	120632	Neethling vaccine LW 1959	LSDV/Russia/Saratov/2017	5.09E-06	8.91E-07	-	#######	#######	-
15	128301	128701	Neethling vaccine LW 1959	KZ-Kostanay-2018	-	3.69E-06	#######	-	-	#######
16	135316	139595	Neethling vaccine LW 1959	155920/2012	9.96E-60	5.96E-55	#######	#######	#######	#######
17	140217	144342	Neethling vaccine LW 1959	KSGP 0240	7.93E-15	1.12E-10	#######	#######	#######	#######
18	148831	150923	LSDV/Russia/Udmurtiya/2019	LSDV/Russia/Dagestan/2015	2.37E-05	1.50E-04	#######	-	-	#######

## Discussion

Two strains of LSDV were isolated in this study, and their genome characteristics were analyzed after sequencing. Homology analysis showed that the nucleotide similarity of the two isolates LSDV XJ201901 and LSDV FJ2019 was 100% and the global LSDV strains with the highest similarity was LSDV72/PrachuapKhiriKhan/Thailand/2021 (99.99%). Interestingly, the isolates in this study are very similar to LSDV isolated in China, Vietnam and Thailand since 2019. But the strain before 2019 with the highest identity with the two isolates is LSDV/Russia/Udmurtiya/2019 (99.46%), which was vaccine-like strain considered as recombinant strain [18]. It is worth noting that the strain KZ-Kostanay-2018 isolated from Kazakhstan also belongs to vaccine like strain according to homology analysis (Fig. 2). The sequence identity between KZ-Kostanay-2018 and other strains in this paper ranges from 97.44% to 99.42%. It was found that KZ-Kostanay-2018 strain show the highest similarity when compared with LSDV XJ201901 and LSDV FJ2019, which can explain why LSDV XJ201901 was separated from the cattle in Chinese border near Kazakhstan. Subsequent gene recombination analysis confirmed the presence of at least 18 gene recombination events in the isolates LSDV XJ201901 and LSDV FJ2019 in further analysis, of which 2 events were related to KZ-Kostanay-2018. However, the strains that contribute most to gene recombination are strains Neethling vaccine LW 1959 and KSGP 0240 (Fig. 5). The identity between the isolated strains and the global LSDV strains based on RP030 gene ranged from 97.70% to 100%, which are exactly the same as the strains isolated in China, Vietnam and Thailand since 2019. However, it should also be noted that the highest identity with the RP030 gene of the strain before 2019 is 99.67%, which are the six strains including Neethling vaccine LW 1959 and Cro2016 and other four vaccine strains on the same branch. The sequence identity between the isolated strains and the global LSDV strains based on GPCR gene ranged from 95.52% to 100%, which also are the same as the strains isolated in China, Vietnam and Thailand since 2019. The highest sequence identity with the GPCR gene of the strain before 2019 is 99.12%, among which the three strains including KSGP 0240, Kenya and Neethling 2490 have the highest identity. In addition, GTPV and SPPV isolated from China were also used for homology analysis, but the isolates in this paper have less identity with GTPV and SPPV comparing with LSDV. Gene recombination events associated with GTPV and SPPV were also not predicted.

The vaccines used for immune prevention against LSDV are mainly field strains attenuated by cell culture at present. LSDV Neethling live attenuated vaccine is one of the earliest successfully developed vaccines [19]. It can not only prevent the occurrence of LSD, but also play an important role in controlling the outbreak of LSD. Immunization with live Neethling vaccine results in lifelong immunity for most immunized cattle. In addition, the group immunity produced by immunization farms can also provide indirect protection for surrounding cattle farms. However, cows vaccinated with the live Neethling strain developed nodules and symptoms of lower viral load, which resulted in reduced milk production. Vaccine virus strains can also be isolated from animals after vaccination with Neethling vaccine [20]. It indicates that although LSDV Neethling live attenuated vaccine has been attenuated, there are still safety problems [21]. The viral secretion or excretion of vaccinated animals may be one of the important reasons for the recombination of LSDV. Border animal trade and insect vector transmission may also aggravate the transmission and recombination of LSDV [22]. The emergence of recombinant LSDV will make the prevention and control of LSD more difficult. LSD genome is huge and the probability of recombination events is high. The emergence of recombinant virus will make the epidemic situation of LSD more difficult to predict and prevent and also make it difficult to isolate and identify the virus. The recombinant virus leads to more subacute symptoms, which will lead to the decline of production performance of infected animals [1].

LSD can be transmitted in a variety of ways, including contact transmission, insect vector transmission, semen and lactation transmission. Although the attenuated vaccine strain has safety problems, it was considered that vaccine is the most effective measure for the prevention and control of LSD due to the lack of insect vector control means [8, 23]. It is generally believed that vaccines have indeed played an important role in many countries. However, the vaccine like strains isolated from diseased cattle suggest that the attenuated vaccine is prone to recombination and even return to strong virulence. The emergence of vaccine like strains not only leads to side effects such as mild fever, but also leads to typical clinical symptoms and even death of infected cattle. The strain of Neethling vaccine LW 1959, Cro16and the isolated strains in this study are all vaccine like strains, which lead to typical clinical symptom. The isolated vaccine like strains are all recombinant strains caused by KSGP 0240 strain or similar strains at present. There is no recombinant vaccine virus caused by the use of sheep pox vaccine. However, there has been reports of contamination of GTPV in LSDV vaccine vials during production [24], which will pose a more severe challenge to the prevention and control of LSDV. This study mainly discussed the recombination of KSGP 0240-like strain predicted by Mega X, RDP4 and Simplot. It was suggested that the countries and regions immunized with sheep pox should pay attention to the recombination of sheep pox vaccine.

## Conclusion

The virus isolate in this study was LSDV and was identified as a vaccine recombinant strain. The most likely potential parent strains of the two strains in this study are Neethling vaccine LW 1959 and KSGP 0240. The strains in this study are very similar to those isolated in East and Southeast Asia since 2019.

## Materials and methods

### Study design

**Fig. 1:**
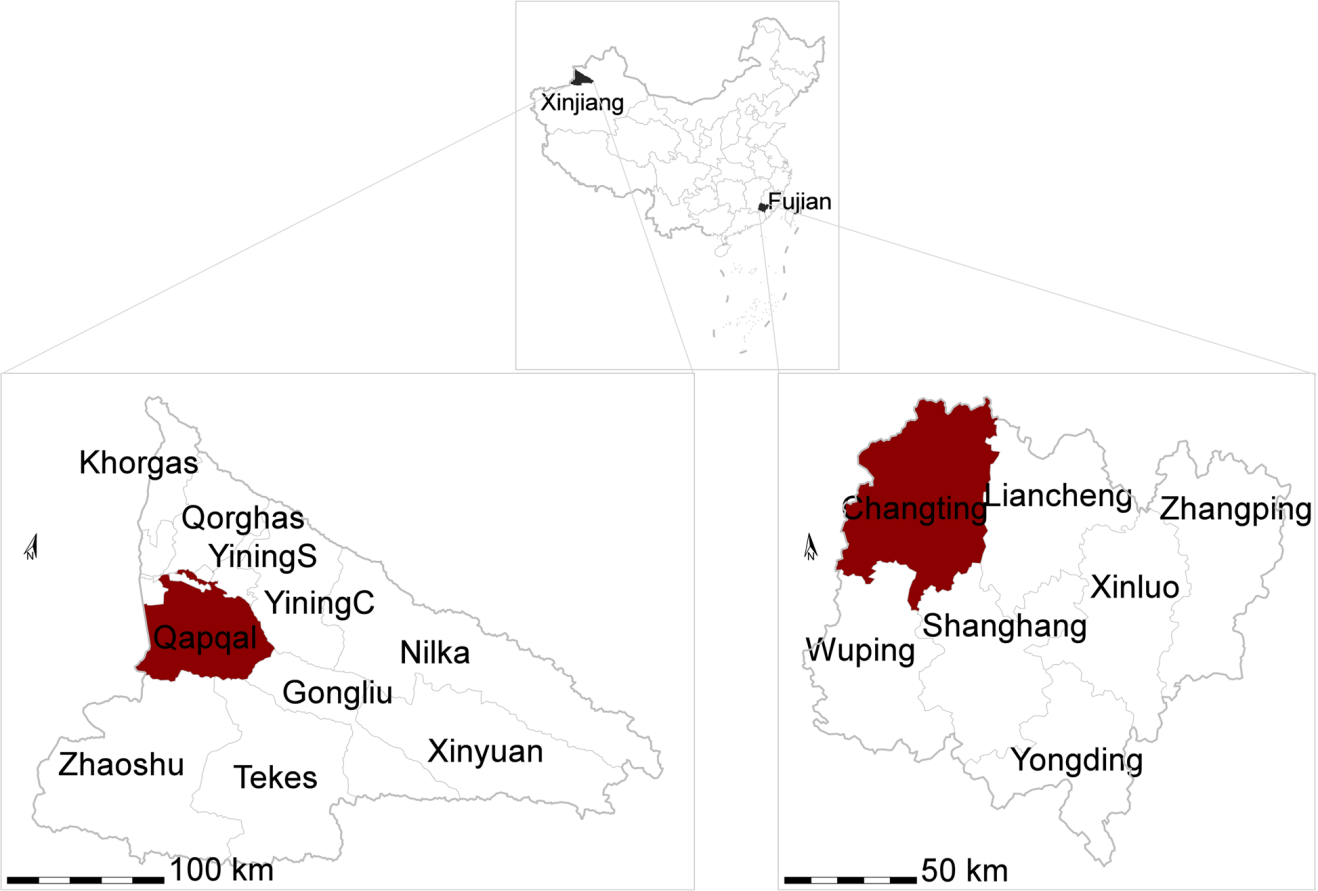
Map of Yili Kazak Autonomous Prefecture of Xinjiang Uygur Autonomous Region and Changting city of Fujian Province

An epidemiological study on LSDV was conducted from 2019 to 2020, and samples from Yili Kazak Autonomous Prefecture in the Xinjiang Uygur Autonomous Region and Changting city of in the Fujian Province were collected (Fig. 1). Figure 1 below shows a map of the Yili Kazak Autonomous Prefecture in the Xinjiang and Changting city in the Fujian Province where the study was conducted.

**Fig. 2:**
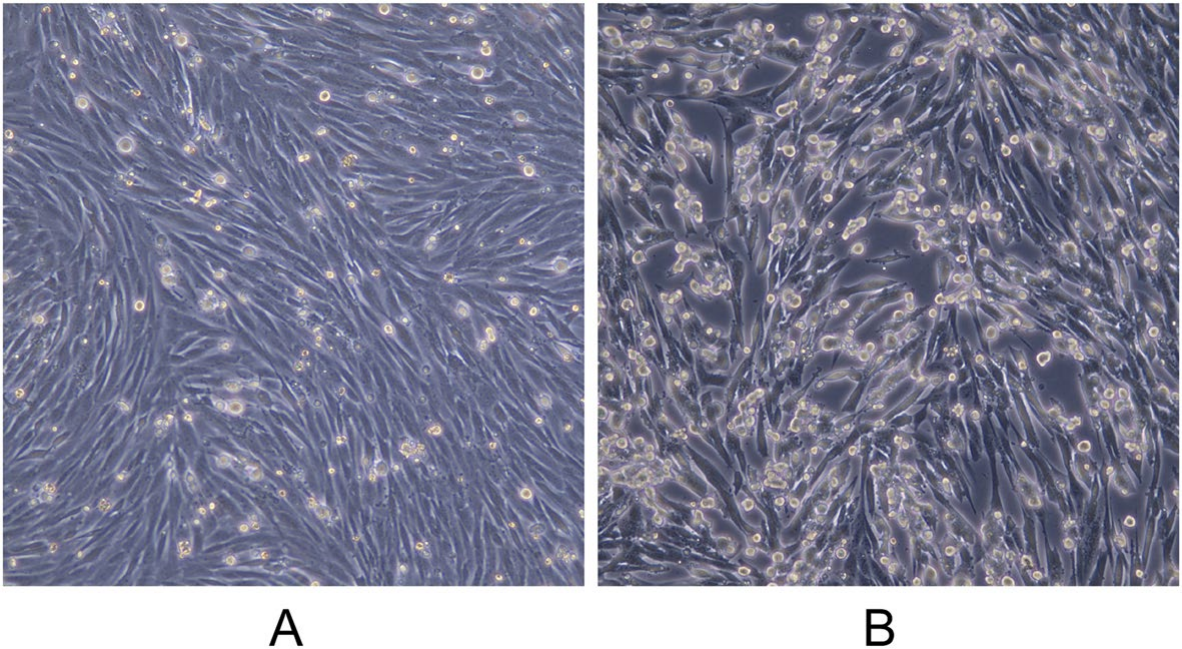
Normal LT cells (**A**) and LT cells infected with LSDV (**B**)

**Fig. 3:**
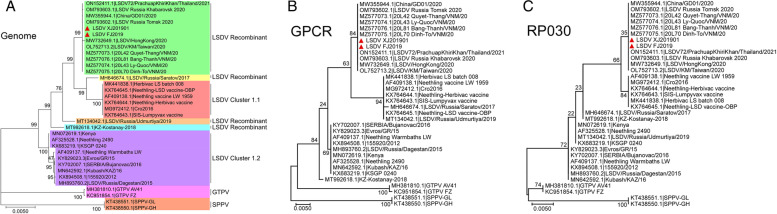
Phylogenetic analysis between isolated strain and the global LSDV strains based on the whole genomic sequences (**A**), GPCR gene (**B**) and RP030 gene (**C**). The isolated strain LSDV XJ201901 and LSDV FJ2019 was marked with red triangles. Homology analysis was performed with Maximum likelihood method using Mega X. The models suitable for genome, GPCR and RP030 identity alignment are calculated. The models suitable for them are GTR (General Time Reversible model), T92 (Tamura-Nei model) and HKY (Hasegawa-Kishino-Yano model) respectively

**Fig. 4:**
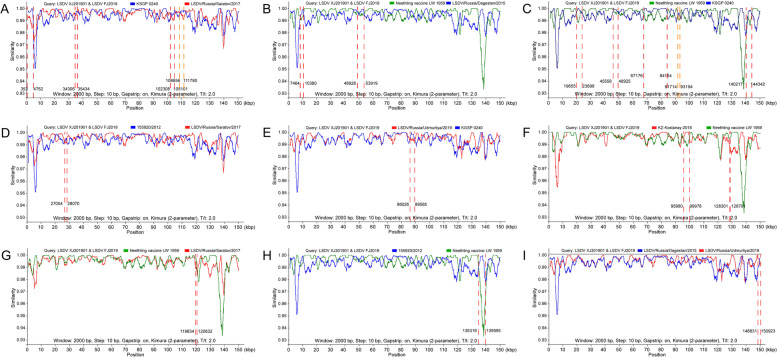
Potential gene recombination events. All global LSDV strains were divided into 3 groups to analyse the gene recombination events involved in LSDV isolates using RDP4 and Simplot. Possible reorganization events and locations are marked in the figure

**Fig. 5:**
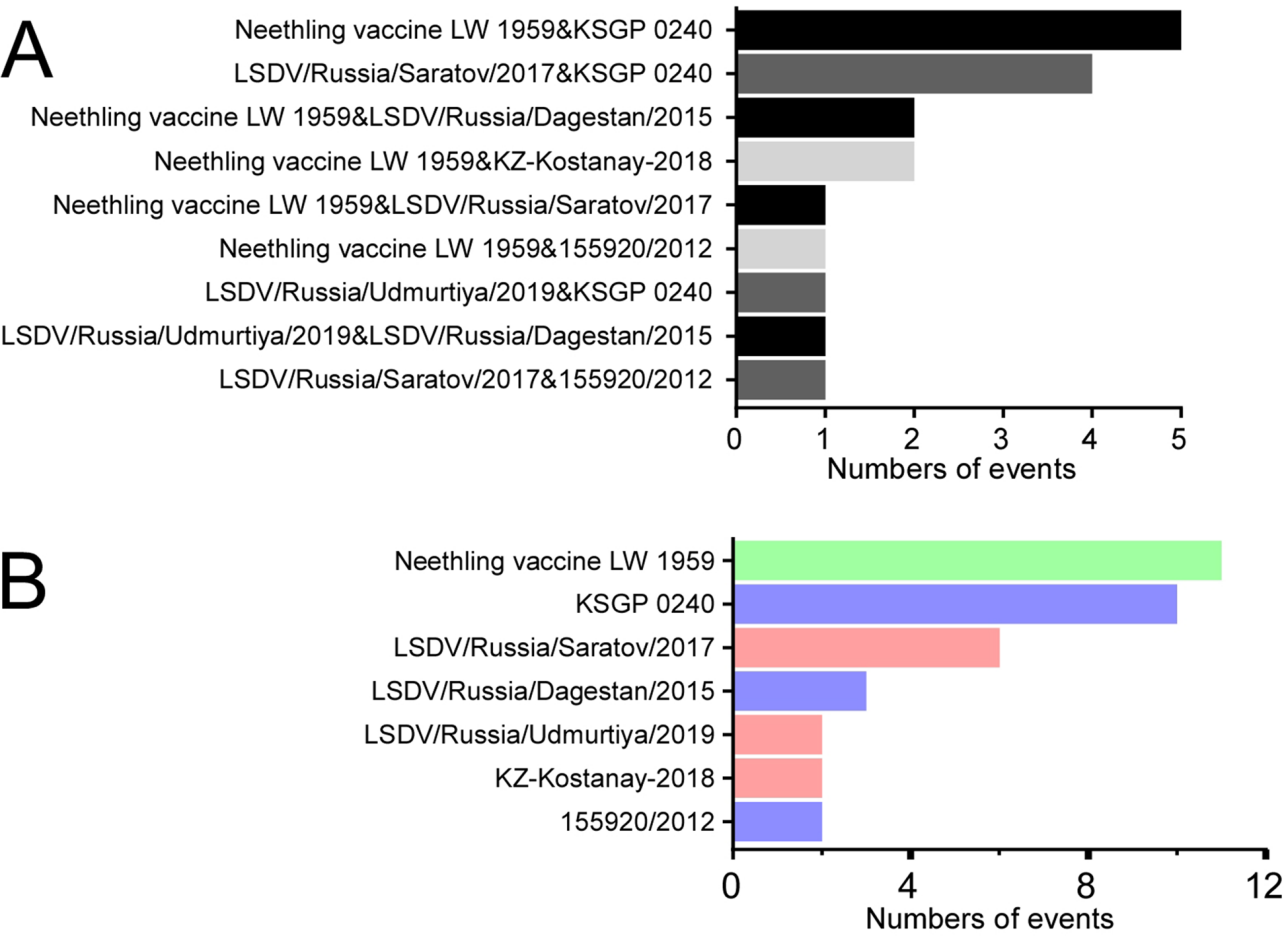
Statistics of gene recombination events. The potential strains involved in gene recombination events are counted (**A**) and shown in the figure and different combinations of strains were labelled with different colour (**B**)

### Sampling of slaughter slabs and cattle

The diseased skin scabbed tissue was collected from diseased cattle in the cattle farm of Qapqal Xibe Autonomous County, Yili, Xinjiang Uygur Autonomous Region and Changting, Fujian. 65 samples were taken from a Yili cattle farm and 11 samples were taken from Changting cattle farm. Each cow is numbered, and the numbered information and biological data of the cow are recorded on paper and folded.

### Sample transport and storage

After collection, scabbed samples were immediately transported to the district veterinary laboratory for temporary storage in a fridge at 4 °C within 2h. Later, samples were transported (in an icebox at 4 °C) to Provincial Department of Agriculture of Xinjiang Uygur Autonomous Region and Fujian Province and stored in a − 20 °C freezer. The transportation of samples was approved by Xinjiang Uygur Autonomous Region and Fujian Provincial Department of Agriculture. The samples were packaged in an ice box containing ice packs at 4 °C, where upon arrival they were placed in a − 80 °C freezer for subsequent DNA extraction.

### DNA extraction and real-time PCR (qPCR)

The samples were thawed in the biosafety level II laboratory at room temperature. A small part of the sample was cut off and put into a 2ml EP tube. After adding 500 μL PBS, it was fully homogenized Lysing Matrix Tubes (MP Biomedicals) using 3D Cryogenic Grinder (Shanghai Jingxin Industrial Development). The supernatant was separated by centrifugation which was used for DNA extraction. DNA extraction was performed by Magnetic Viral DNA/RNA Extraction Kit (Tianlong, China) according to the manufacturer’s protocol. A real-time (quantitative) PCR was performed using the ChamQ Geno-SNP Probe Master Mix (Vazyme, China). qPCR was performed in a Roche® LightCycler 480PCR System. Primers and probes refer to national standard code for Chinese characters (Table 1) in ‘Diagnostic techniques for Lumpy skin disease’ (GB/T 39602-2020).

A qPCR master mix containing 3.5 μL double distilled water, 1.25 μL of 10 μM Forward, 1.25 μL of 10 μM reverse primers, 1.5 μL of 10 μM probe and 12.5 μl of ChamQ Geno-SNP Probe Master Mix (Vazyme, China) was completely mixed by tapping the tube and a quick short spin for one reaction. The components of the master mix were adjusted to suit the number of samples. The contents of the master mix tube were mixed thoroughly and dispensed 20 μl to each labeled sample and control tubes. An DNA template of 5 μl was then dispensed to each tube with a master mix. The tubes were placed in a Roche® LightCycler 480PCR System and the program which includes apre-heating at 95 °C for 5 min, denaturation at 95 °C for 30 seconds and annealing at 60 °C for 15 seconds was started. This was repeated for 40 cycles with preheating occurring just once. The total DNA was extracted by Magnetic Viral DNA/RNA Extraction Kit (Tianlong, China) according to the instruction manual.

### Data analysis

Strain identification was determined by plotting amplification curves of fluorescence signal detected versus cycle threshold values (Ct). Cycle threshold values of ≤37 were considered positive and Ct value > 37 were taken as negative.

### Virus isolation and identification

The Lamb testis (LT) primary cells were preserved in our laboratory. The cell culture medium was MEM (Gibco, US) containing 10% FBS (Thermo, US) and 2% penicillin-streptomycin. LT cell culture medium for virus isolation contained 10% (V/V) penicillin-streptomycin. All live virus-related operations involved in this study were performed in a biosafety level III laboratory.

The aseptically collected diseased bovine skin tissue samples were grounded and resuspended with PBS containing 10% penicillin-streptomycin. After centrifugation, the supernatant was taken and inoculated onto LT cells. After 2 hours, the cells were cultured with MEM containing 10% FBS until cytopathic effect (CPE) appeared. Then the cell culture was harvested, repeatedly frozen and thawed for 3 times, and then centrifuged to collect the supernatant as the virus stock solution.

### Genomic sequencing

The extracted total DNA genome was handed over to Beijing Novogene for next-generation sequencing. And the sequence is spliced using the software of Geneious prime 2022.0.1 with the strain of China/GD01/2020 (Genebank: MW355944) as reference genome.

### Phylogenetic analysis

The reference sequences of LSDV were downloaded from NCBI and the sequence alignment of sequenced strains and the reference sequences was conducted by Mega X with the Maximum likehood method and General Time Reversible model with genome, Hasegawa-Kishino-Yano model with RPO30 gene and Tamura-Nei model with GPCR gene. Phylogeny test is performed using Bootstrap method for replicating 1000 times.

### Recombination analysis

The software of RDP4 was used to align and analyse the recombination of LSDV in this study with other reference sequences. The software of Simplot was used to find the potential recombinant events.

## Data Availability

All the samples and PCR products for this paper are stored at Biosafety level III Laboratory of China Animal Health and Epidemiology Center, Qingdao, China and can be obtained upon request. The whole genome sequences of LSDV XJ201901 and LSDV FJ2019 have been submitted to NCBI, one of which is named LSDV XJ201901 (Genebank: OM984485) and the other is named LSDV FJ2019 (Genebank: OM984486). ​ Lumpy skin disease virus strain LSDV XJ201901, complete genome - Nucleotide - NCBI (nih.gov) UNVERIFIED: Lumpy skin disease virus strain LSDV FJ2019, complete genome - Nucleotide - NCBI (nih.gov)
